# Human adipose liquid extract induces angiogenesis and adipogenesis: a novel cell-free therapeutic agent

**DOI:** 10.1186/s13287-019-1356-0

**Published:** 2019-08-14

**Authors:** Yunfan He, Jing Xia, Hsinkai Chen, Liangyue Wang, Chengliang Deng, Feng Lu

**Affiliations:** 1 Department of Plastic Surgery, Nanfang Hospital, Southern Medical University, 1838 Guangzhou North Road, Guangzhou, 510515 Guangdong China; 2 Department of Plastic Surgery, Affiliated Hospital of Zunyi Medical College, Zunyi, 563100 Guizhou China

**Keywords:** Growth factors, Cell free, Angiogenesis, Adipogenesis, Wound healing, Tissue regeneration

## Abstract

**Background:**

Taking advantage of cellular paracrine mechanisms, the secretome of adipose-derived stem cells (ADSCs) and adipose tissue has been demonstrated to induce tissue repair and regeneration in various ischemic and impaired conditions. However, these cell-based therapies have been hindered by issues, such as inherent safety and cost-efficiency for clinical applications. In this study, we prepared a liquid cell-free extract from human adipose tissue [adipose liquid extract (ALE)] and evaluated its potential therapeutic efficacy.

**Methods:**

ALE was prepared from human subcutaneous adipose tissue using a rapid and physical approach, and the protein components in ALE were identified using mass spectrometry analysis. In vivo, the therapeutic effect of this agent was investigated on wound healing in C57BL/6 mice, and wound healing rate, vessel density, and neo-adipocyte formation in wounded skins were measured at days 3, 7, 11, and 14. In vitro, the effect of ALE on the viability of human ADSCs, tube formation of human umbilical vein endothelial cells (HUVECs), and adipogenic differentiation of ADSCs were tested.

**Results:**

The results demonstrated that ALE contained a variety of growth factors and did not affect cell viability. ALE-treated wounds exhibited accelerated wound healing with increased vessel density and formation of neo-adipocytes compared to that of control wounds. Moreover, when added as a cell culture supplement, ALE effectively induced tube formation of HUVECs and lipid accumulation in ADSCs. ALE-treated ADSCs also exhibited elevated levels of adipogenic gene expression.

**Conclusions:**

ALE is a novel growth-rich therapeutic agent that is cell-free and easy to produce. Besides, it is also able to induce angiogenesis and adipogenesis both in vitro and in vivo, thus indicating that it could be used for wound repair and soft tissue regeneration.

## Background

Tissue repair and regeneration are complex biological processes that occur throughout human life. Therapeutic induction of damaged tissue/organs requires provision of instructive bioactive signals, and the most crucial among these signals are growth factors [1]. Growth factors are important biomolecules that instruct cell behavior (for example, cell migration, survival, adhesion, proliferation, and differentiation) and guide tissue regenerative process [2–4]. Till date, employment of exogenous growth factor products as therapeutic agents for clinical use is limited so far as the ease/cost of production, specificity of action, and suboptimal efficacy are concerned [5]. Hence, there is an urgent need for new technologies and strategies that may render growth factors more amenable to clinical treatment.

Taking advantage of paracrine secretory function of cells, developments in growth factors therapies have been reported to make use of cell-derived secretome, which is the complex set of biomolecules secreted during short-term culture in vitro [6]. The cell-derived secretome technology is a rapidly advancing field and has shown a successful outcome in stimulating tissue repair and regeneration both in vitro and in vivo [7–10]. Furthermore, a secretome extract harvested from cultured adipose tissue was shown to contain various angiogenic and adipogenic factors that were able to induce angiogenesis and adipogenesis in vitro [11]. Although the secretome of these cultured cells/tissue exhibits efficient therapeutic efficacy, there are several hurdles that are needed to be addressed before translating this therapy from bench-to-bedside. Examples of these drawbacks include isolation of cells using enzymatic digestion that increases the risk of biological contamination, the secretome harvested from cultured cells/tissues requires considerable cell expansion, specific laboratory equipment, and good manufacturing practice, which in turn increase the financial burden [12].

Adipose tissue has currently gained significant importance since it serves as an active endocrine organ and an abundant source of biologically active substances [13–15]. The study by Tonnard et al. demonstrated that the human lipoaspirates can be physically emulsified into a therapeutic liquid suspension (e.g., Nanofat) for skin rejuvenation [16]; recent studies have demonstrated that Nanofat contains a number of growth factors that are able to induce angiogenesis in vivo [17, 18]. Our research team utilized a similar physical method to process human lipoaspirates [19]. After centrifugation, we found that the processed lipoaspirates were separated into four parts (from top to bottom: lipid portion, lipid-devoid adipose tissue, liquid portion, and cell/tissue debris). The lipid-devoid adipose tissue, namely, extracellular matrix/stromal vascular fraction gel (SVF-gel), was found to have therapeutic ability in inducing angiogenesis and adipogenesis in vivo [20–24]. Unfortunately, the study of the liquid portion acquired from these physically processed lipoaspirates has been ignored. We speculated that this adipose tissue-derived liquid portion may contain a variety of bioactive factors that may exhibit therapeutic ability in regenerative medicine.

In this study, we described a novel method to obtain this adipose tissue-derived liquid portion, the “adipose liquid extract” (ALE), and evaluated its therapeutic effect on wound healing in mice. The protein components and osmotic pressure of ALE were measured, and the effect of ALE on cell viability was evaluated. More importantly, the effect of ALE on angiogenesis and adipogenesis was evaluated both in vitro and in vitro.

## Methods

### Ethics statement

All the procedures including animal studies were approved by Southern Medical University Institutional Review Board and the Nanfang Hospital Animal Ethics Committee and conducted in accordance with the ethical standards of the National Health and Medical Research Council (China).

### ALE preparation and osmotic pressure measurement

**Fig. 1:**
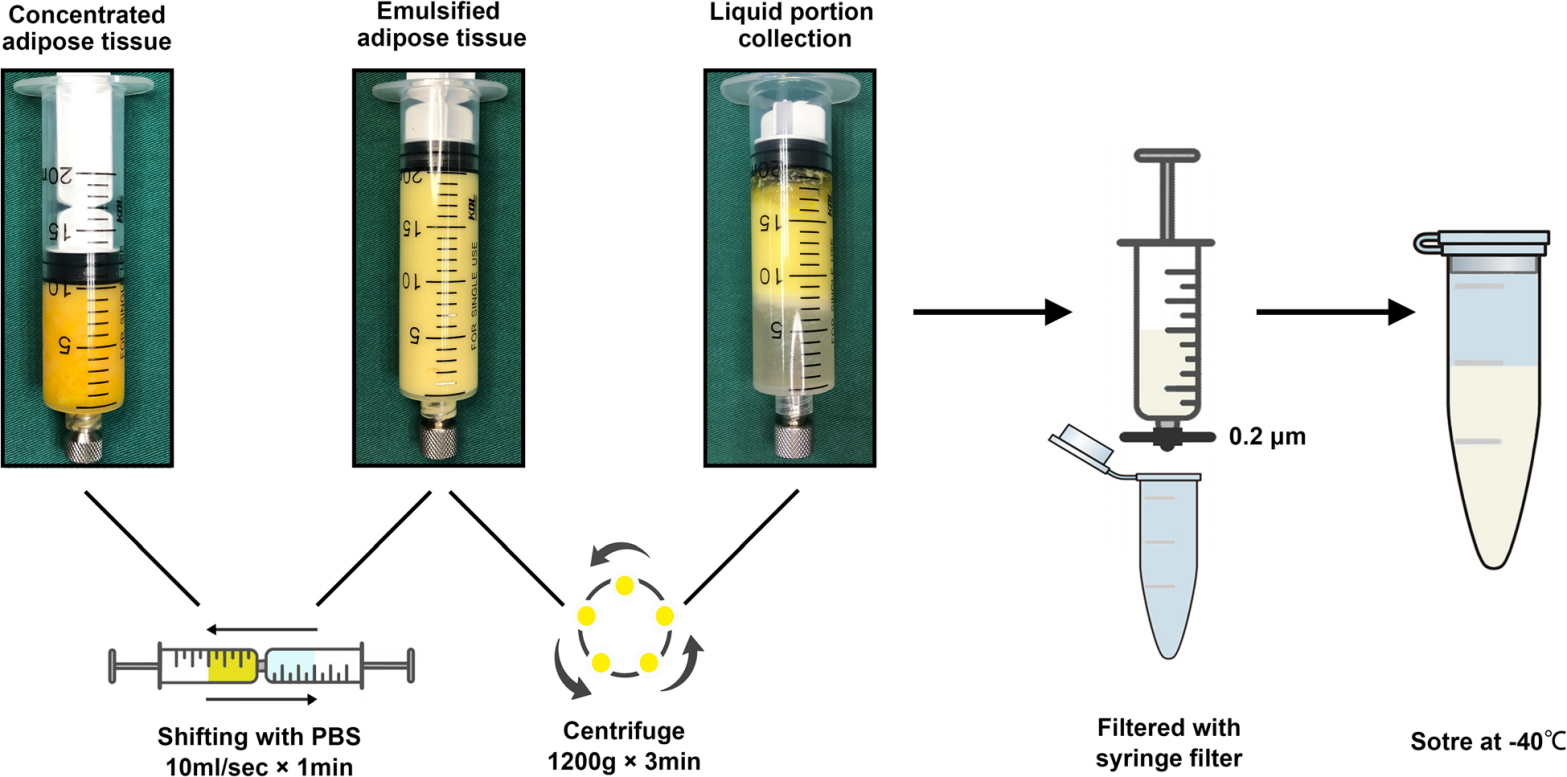
Schematic overview of ALE preparation

Human adipose tissues were harvested from female patients undergoing abdominal liposuction in the Plastic Surgery Department of Nanfang Hospital, after taking their informed consent. Detailed procedure of ALE preparation is shown in Fig. 1. First, the harvested adipose tissue was allowed to stand for 10 min in ice water. The liquid portion was discarded and adipose tissue layer collected. The obtained adipose tissue was then washed with phosphate-buffered saline (PBS) and centrifuged at 1200*g* for 3 min to remove the remaining blood constituents. The concentrated adipose tissue was mixed with an equal volume of PBS and then physically emulsified by repeated transfer between two 10-mL syringes connected by a female-to-female Luer-Lock connector with an internal diameter of 1.4 mm. Transfer of the adipose tissue was carried out for 1, 3, 5, 7, or 10 min at a constant rate (10 mL/s). After centrifugation at 2000*g* for 5 min, the liquid portion (ALE) from emulsified adipose tissue was collected and passed through a 0.20-μm syringe filter (Xiboshi, DIONEX, USA) to remove cell and tissue debris. The osmotic pressure of ALE was measured using Fiske® 210 Micro Osmometer and the sample stored at − 40 °C until use. ALE emulsified for 1 and 10 min were subjected to mass spectrometric analysis to investigate the effect of time variations on protein yield of ALE. All the five ALE samples were used for cell viability assay and osmotic pressure measurements, while only the ALE emulsified for 1 min was used for all the remaining experiments.

### Mass spectrometry identification and database searches

The ALE samples were first digested by trypsin for subsequent liquid chromatography tandem mass spectrometry (LC-MS/MS) analysis as described before [25]. The tryptic peptide products were then separated by reverse-phase liquid chromatography using nano LC system (DIONEX Thermo Scientific). Label-free LC-MS/MS identification was subsequently conducted using a Q Exactive Plus (Thermo Fisher Scientific, Marietta, OH, USA) equipped with a self-packed column (Thermo Fisher Scientific, Acclaim PepMap RSLC 50 μm × 15 cm, nano viper, P/N164943) at a flow rate of 300 nL/min according to previous study [26]. Label-free experiments’ data was analyzed using MaxQuant program with high-resolution instruments supported by Andromeda as a database search engine for peptide identification [27]. Label-free quantitation (LFQ) was performed as previously described [28]. LFQ intensity values were used for protein quantification, and protein information was searched against the human database (Uniprot_HomoSapiens_161584_20180123).

Gene Ontology (GO) analysis of ALE was performed to classify all identified proteins into two categories (biological process and molecular function) using Blast2GO v.2 software [29] and web database (www.geneontology.org). Functional annotation of proteins related to angiogenesis and adipogenesis was identified. Ingenuity Pathway Analysis (IPA) analysis of identified proteins in ALE was performed to obtain protein location information through a web database (https://www.qiagenbioinformatics.com).

### Growth factor measurement

High sensitivity enzyme-linked immunosorbent assay (ELISA) kits (R&D Systems, Minneapolis, MN, USA) was used to quantify the levels of basic fibroblast growth factor (bFGF), epidermal growth factor (EGF), transforming growth factor-β1 (TGF-β1), and vascular endothelial growth factor (VEGF), hepatocyte growth factor (HGF), and platelet-derived growth factor (PDGF) in ALE according to the manufacturer’s instructions.

### Cell viability assay

Primary human adipose-derived stem cells (ADSCs) were isolated from adipose tissue as described previously [30, 31]. Briefly, fresh adipose tissue was washed and digested in 0.075% collagenase solution for 40 min on a shaker at 37 °C. The digested tissue was centrifuged at 180*g* for 5 min and then filtered to remove large debris. Next, the cellular pellet (SVF) was re-suspended in an erythrocyte lysis buffer and centrifuged at 180*g* for another 5 min. Finally, the SVF was cultured in human ADSC complete growth medium (HUXMD-90011, Cyagen, China). Passage 3 ADSCs were used in subsequent experiments.

ADSCs were treated with the five ALE samples to assess the effect of ALE on cell viability. Briefly, the ADSCs (5 × 10^3^ cells/well) were incubated in a 96-well microplate at 37 °C for 24 h to permit cell adhesion and were later treated with each of the five ALE samples or PBS (control group). The relative cell viability present at the 12, 24, and 36 h time points was estimated using a Cell Counting Kit-8 assay kit (CCK-8, Dojindo, Japan) according to the manufacturer’s instructions. Absorbance was measured using a plate reader at 450 nm, and the OD values in different groups were used to analyze cell viability. These experiments were repeated three times.

### Angiogenic induction ability of ALE in vitro

Human umbilical vein endothelial cells (HUVECs) were purchased from the American Type Culture Collection (ATCC, Rockville, MD, USA) and cultured in endothelial cell medium (1001, ScienCell, USA). Tube formation assays were conducted by treating HUVECs with ALE to assess the angiogenesis-inducing ability of ALE. In brief, frozen Matrigel (356230; Becton, Dickinson & Co., Franklin Lakes, NJ, USA) was thawed overnight at 4 °C, and all experimental equipment were cooled to − 20 °C before use. The Matrigel (diluted 1:1 in PBS) was transferred to a 24-well plate and solidified by incubation at 37 °C and 5% CO_2_ for 30 min before seeding of the cells. HUVECs (5 × 10^5^ per well) were then seeded on the Matrigel and treated with 2 mL medium (1:1, ALE to endothelial cell basal medium). HUVECs treated with angiogenic factors [20 ng/mL vascular endothelial growth factor (VEGF) and 5 ng/mL basic fibroblast growth factor (bFGF)] served as a positive control, while cells treated with 2 mL medium (1:1, PBS to endothelial cell medium) were used as a negative control. After 3, 9, and 20 h, tube formation of HUVECs was observed and photographed using a Nikon E200 microscope (Nikon Corp., Tokyo, Japan). Angiogenesis is evaluated by counting the number of tubular structures from six fields of each well. Tube formation assays were repeated three times.

### Adipogenic induction ability of ALE in vitro

ADSCs were treated with ALE to assess the adipogenic potential of ALE. In brief, ADSCs (1 × 10^5^ per well) were plated on 6-well plates and cultured overnight at 37 °C and 5% CO_2_. The next day, cells in each well were rinsed and treated with 2 mL medium (1:1, ALE to human ADSC complete growth medium). ADSCs treated with adipogenesis kit (HUXMD-90031, Cyagen Biosciences, Guangzhou, China) served as the positive control, and cells treated with 2 mL medium (1:1, PBS to human ADSC complete growth medium) were used as the negative control. The medium in all the groups was changed every 3 days. Adipogenic differentiation of ADSCs was examined for lipid accumulation by Oil Red O (ORO; Sigma-Aldrich, USA) staining. Cells were photographed using a Nikon E200 microscope and collected for gene expression analysis at days 4, 7, 14, 21, and 28. Adipogenic induction assays were repeated three times.

### Therapeutic effect of ALE in vivo

The therapeutic effect of ALE in vivo was evaluated by the application of ALE in a wound healing model of mice. Female C57BL/6 mice, 4- to 6-week-old (*n* = 20), were purchased from the Southern Medical University Laboratory Animal Center and were maintained in microisolator cages at the Animal Experiment Center of Nanfang Hospital. For wound healing experiments, mice were anesthetized and two circular full-thickness excisional skin wounds of 8-mm radius were made on the dorsum of mice. A silicone ring was then placed around the perimeter of the wound and secured with 6-0 sutures to prevent wound contraction. Wounds were randomly treated either with 200 μL ALE or 200 μL PBS (control group) in each mouse. After the treatments, the wounds were covered with Tegaderm sterile dressing (3 M Healthcare, St Paul, Minn.). Treatments were administered to the wounds every 2 days, and sterile dressings were changed every day. Pictures of the wounds were taken, and the skins around the wounds as well as health skins were harvested for further analysis on days 3, 7, 11, and 14 after surgery. The wound area was quantified using ImageJ software, and the wound healing was expressed as follows: residual wound area/original wound area × 100.

### Histological assessment and immunohistochemical staining

Fresh skin samples were fixed in formalin for histological assessment and immunohistochemical staining. Briefly, formalin-fixed samples were embedded and sliced into 5-μm sections. For histological assessment, tissue sections were deparaffinized in xylene, rehydrated through graded alcohol in phosphate-buffered saline (PBS), and then stained with a hematoxylin-eosin (HE) working solution. For immunohistochemical analysis, sections were dewaxed and rehydrated, then incubated in 3% H_2_O_2_ to block endogenous peroxidase. Immunohistochemical staining was performed using antibodies against CD31 (1:25, ab28364, Abcam, Cambridge, UK) and perilipin (1:500, GP29; Progen, Heidelberg, Germany), followed by secondary antibody. Angiogenesis and adipogenesis were evaluated by counting the number of brown-labeled vessel-like structures and brown-labeled adipocytes, respectively, from six fields of each slide.

### Quantitative reverse-transcription polymerase chain reaction (qRT-PCR)

Total RNA in ADSCs was extracted and measured using qRT-PCR. Primers used for adipogenesis-specific genes were from peroxisome proliferator-activated receptor gamma (*PPAR-γ*) and CCAAT/enhancer-binding protein alpha (*CEBP-α*), master regulators for the induction of adipogenic differentiation. RNA was isolated from the samples using Trizol and SYBR Green quantitative PCR SuperMix (Thermo Fisher Scientific, Marietta, USA), followed by reverse transcription. The complementary DNA samples were measured using customized TaqMan Array Plates (Thermo Fisher Scientific, Marietta, USA). Relative expressions were calculated by the cycle threshold method using GAPDH as an endogenous reference gene.

### Statistical analysis

Data are expressed as mean ± SD. Results were analyzed using SPSS 22.0 software. An independent *t* test and least significant difference post hoc analysis were used to compare two and three groups at a single time point, respectively, and one-way analysis of variance (ANOVA) was used to compare groups at all time points. OD values from different ALE samples were subjected to one-way ANOVA followed by Tukey’s multiple comparison test to evaluate the cell viability with ALE treatment. Values of *p* < 0.05 were considered statistically significant.

## Results

### Proteomic profiles and functional categories of identified proteins in ALE

**Fig. 2:**
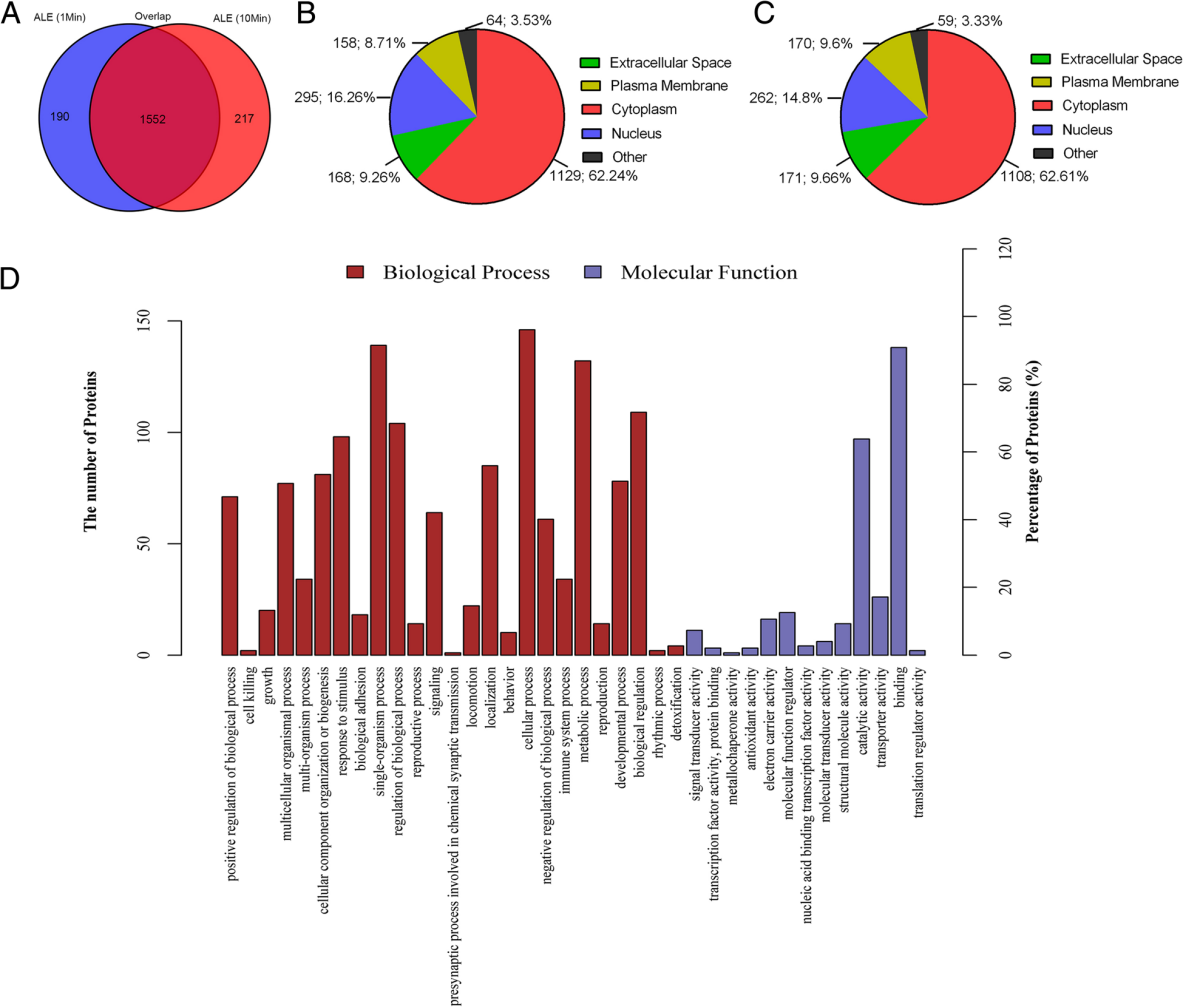
Mass spectrometry-based quantitative proteomic analysis of ALE and classification of identified proteins. **a** Expression profiling of proteins in 1 min ALE and 10 ALE. **b** Subcellular distribution of proteins in 1 min ALE. **c** Subcellular distribution of proteins in 10 min ALE. **d** Biological processes and molecular function categories of identified proteins in ALE

ALE samples (1 min processing time and 10 min processing time) were analyzed by label-free LC-MS/MS and Maxquant software to acquire their quantitative proteomic profiles. The mass spectrometry analysis showed that totally 1975 human proteins were reliably quantified (1742 in 1 min processed ALE and 1769 in 10 min processed ALE). Interestingly, over 87% of the identified proteins in the two groups overlapped with one another (1552 out of 1742 in the 1 min processed group and 1552 out of 1769 in the 10 min processed group) (Fig. 2a). IPA analysis showed that the majority of ALE proteins were from the cytoplasm, followed by the nucleus, and finally from the extracellular space and plasma membrane (Fig. 2b, c).

The GO analysis of biological processes indicated that the proteins in ALE were mainly involved in the cellular process, biological regulation, and metabolic process. In addition, the three most abundant classes of the molecular functions in ALE were binding, catalytic activity, and molecular function regulators (Fig. 2d). Functional annotation revealed that 67 proteins were involved in angiogenesis (Table 1) and 25 proteins were involved in adipogenesis (Table 2). Elisa assay was carried out to further measure the level of growth factors in ALE. High levels of growth factors (bFGF, EGF, TGF-β1, VEGF, HGF, and PDGF) were detected in the ALE, and the expression level of each factor are presented in Table 3.

**Table 1:** Angiogenesis-related proteins identified in ALE

Angiogenesis-related proteins	Gene	Angiogenesis-related proteins	Gene
Lysosomal Pro-X carboxypeptidase	PRCP	Caveolin 1	CAV1
Glutathione peroxidase 1	GPX1	NID2 protein	NID2
Fibrinogen alpha chain	FGA	Aminoacyl tRNA synthase complex-interacting multifunctional protein 1	AIMP1
ATP synthase subunit beta	HEL-S-271	Collagen alpha-1(VI) chain	COL6A1
Heparan sulfate proteoglycan 2	HSPG2	Alpha-parvin	PARVA
Apolipoprotein D	APOD	Melanoma cell adhesion molecule	MCAM
Phosphoinositide phospholipase C	PLCD1	Myosin, heavy polypeptide 9	MYH9
Focal adhesion kinase 1	PTK2	Cadherin-13	CDH13
Neuronal cell adhesion molecule	NRCAM	Extracellular matrix protein 1	ECM1
Thymidine phosphorylase	TYMP	Alpha-crystallin B chain	CRYAB
Leucyl and cystinyl aminopeptidase	LNPEP	Programmed cell death 6	PDCD6
cAMP-dependent protein kinase catalytic subunit gamma	PRKACG	High mobility group protein B1	HMGB1
Glucose-6-phosphate isomerase	GPI	High-mobility group box 2	HMGB2
FN1 protein	FN1	Myeloid-derived growth factor	MYDGF
Mitogen-activated protein kinase	MAPK14	Platelet factor 4	PF4
Upstream-binding protein 1	UBP1	Sorcin	SRI
Collagen, type XVIII, alpha 1, isoform CRA_d	COL18A1	Protein kinase cAMP-dependent type I regulatory subunit alpha	PRKAR1A
GDP-fucose protein O-fucosyltransferase 1	POFUT1	Annexin A1	ANXA1
Collagen alpha-1(XV) chain	COL15A1	Related RAS viral (R-ras) oncogene homolog, isoform CRA_a	RRAS
Myosin-10	MYH10	Ras-related protein Ral-A	RALA
Protein kinase, cAMP-dependent, catalytic, alpha	PRKACA	Epididymis luminal protein 55	HEL55
Integrin beta-1	ITGB1	Annexin A4	ANXA4
Periostin isoform thy6	POSTN	Annexin A7	ANXA7
Chloride intracellular channel protein 2	CLIC2	Epididymis secretory protein Li 102	HEL-S-102
Transforming growth factor beta 1	TGFBI	Dimethylarginine dimethylaminohydrolase 1	DDAH1
Nucleolin	NCL	RAP1A, member of RAS oncogene family	RAP1A
Chloride intracellular channel protein	CLIC4	Chymase A1	CMA1
Epididymis secretory sperm binding protein Li 62p	HEL-S-62p	Endoplasmic reticulum aminopeptidase 1	ERAP1
Complement component C6	C6	Annexin A3	ANXA3
Cytoplasmic protein NCK1	NCK1	Complement C5	C5
Annexin A11	ANXA11	Annexin A5	HEL-S-7
Annexin 6	ANXA6	Epididymis secretory sperm binding protein	RHOA
Collagen alpha-2(VI) chain	COL6A2	Ras-related protein R-Ras2	RRAS2
Chloride intracellular channel protein	CLIC1

**Table 2:** Adipogenesis-related proteins identified in ALE

Adipogenesis-related proteins	Gene	Adipogenesis-related proteins	Gene
Aldehyde dehydrogenase 6 family, member A1, isoform CRA_b	ALDH6A1	Leucine-rich alpha-2-glycoprotein 1	HMFT1766
Glutathione peroxidase 1	GPX1	Adipogenesis associated Mth938 domain containing	AAMDC
Solute carrier family 2 member 1	SLC2A1	Adipogenesis regulatory factor	ADIRF
Glutathione peroxidase 1 isoform A	GPX1	Adiponectin B	C1QC
ERO1-like protein alpha	ERO1A	Sulfotransferase A4	SULTA4
Adiponectin, C1Q and collagen domain containing	ADIPOQ	Protein kinase catalytic subunit gamma	PRKACG
Epididymis secretory protein Li 104	FABP4	Alpha-ketoglutarate-dependent dioxygenase FTO	FTO
C-terminal-binding protein 1	CTBP1	AKT serine/threonine kinase 2	AKT2
Protein kinase catalytic subunit alpha	PRKACA	Hepatocyte growth factor-regulated tyrosine kinase substrate	HGS
Proteasome subunit beta 8	PSMB8	Nidogen 2	NID2
Endoplasmic reticulum aminopeptidase 1	ERAP1	Mitogen-activated protein kinase 14	MAPK14
Solute carrier family 2 member 4	SLC2A4	Sulfotransferase	hCG_1993905
Proteasome subunit beta type-5	PSMB5

**Table 3:** Growth factor concentrations (pg/ml) in ALE

Growth factors	bFGF	EGF	TGFβ-1	VEGF	HGF	PDGF
Mean ± SD	690.99 ± 135.39	67.68 ± 27.78	1430.34 ± 383.51	436.70 ± 174.27	470.07 ± 169.20	169.71 ± 40.02

### ALE did not affect cell viability

**Fig. 3:**
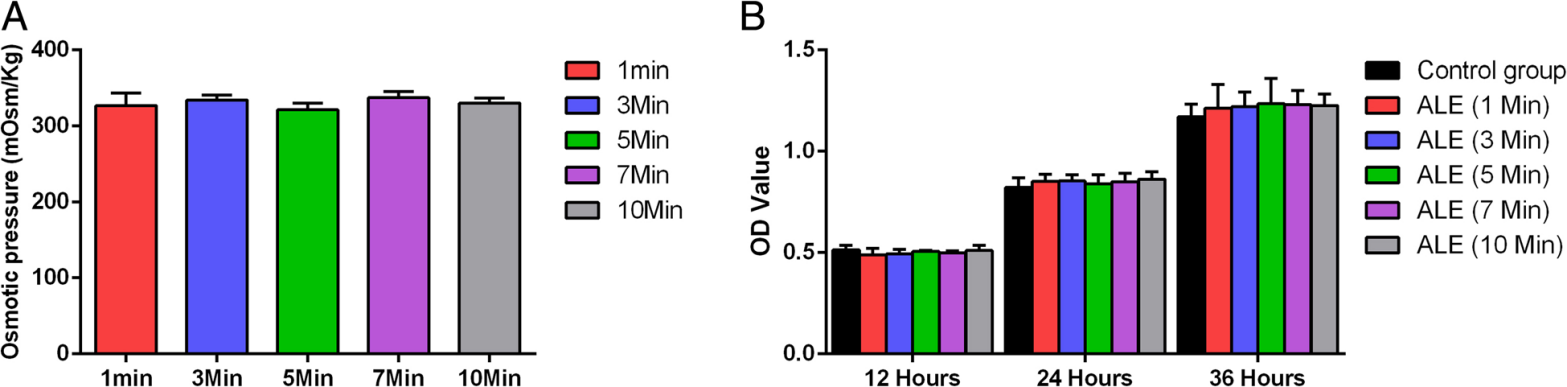
Osmotic pressure measurement and cell viability assay. **a** Osmotic pressure measurement of five ALE samples. **b** Cell viability assessment of ADSCs with five ALE samples’ treatment

Osmotic pressure measurement showed that there were no significant differences among all the five ALE samples and the values remained in the normal physiological range (280–310 mOsm/kg) (Fig. 3a).

The viability of ALE-treated ADSCs was evaluated at 12, 24, and 36 h. Although the ALE samples seemed to lower the cell viability than that in the control group at 12 h, the difference was not significant. Similarly, we did not observe any significant variation in the viability of ADSCs by treating with all the five ALE samples at 24 and 36 h (Fig. 3b).

### ALE enhanced wound healing in mice

**Fig. 4:**
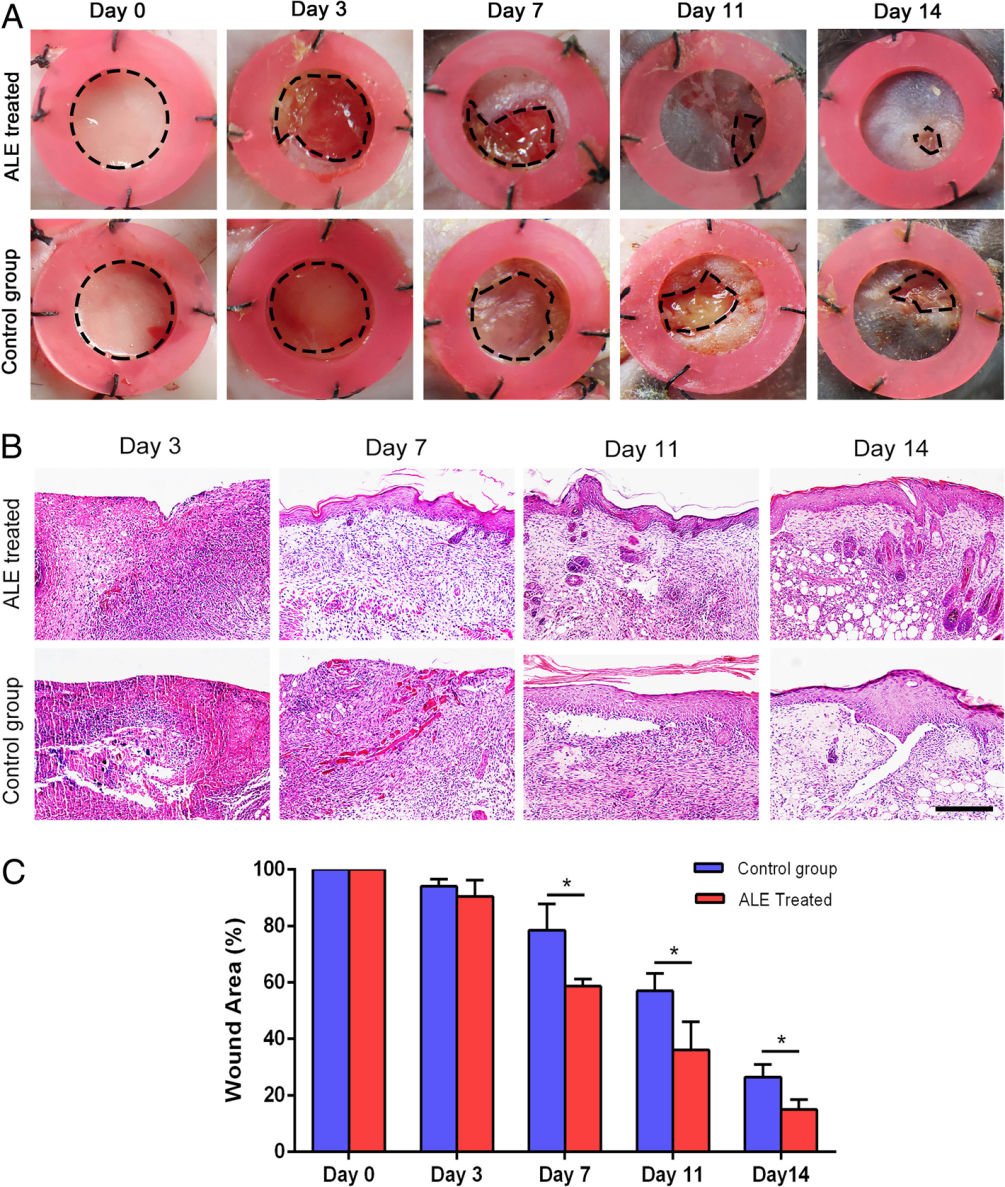
Therapeutic effect of ALE on wounds. **a** Representative images of wound beds treated with either ALE or PBS in mice. **b** HE staining of wounded skins. **c** Quantification of the rate of wound healing. *P < 0.05. Scale bars = 200 μm

Figure 4a shows representative images of the full-thickness wound beds treated with either ALE or PBS in mice. Histological observations showed that inflammatory cell infiltration could be observed in both the groups on day 3. On day 7, the ALE-treated wounds showed re-epithelialization, while almost no re-epithelialization could be observed in the control group. On day 14, wounds in the ALE-treated group exhibited increased skin appendage numbers compared to those in the control group (Fig. 4b).

Statistical analysis of the rate of wound healing revealed that the healing of wounds was significantly faster in the ALE-treated group than in the control group from day 7 to day 14 (Fig. 4c). Moreover, the ALE-treated group nearly achieved complete wound healing by day 14, whereas the control group still had unhealed wounds at that point of time.

### ALE induced angiogenesis in vitro and in vivo

**Fig. 5:**
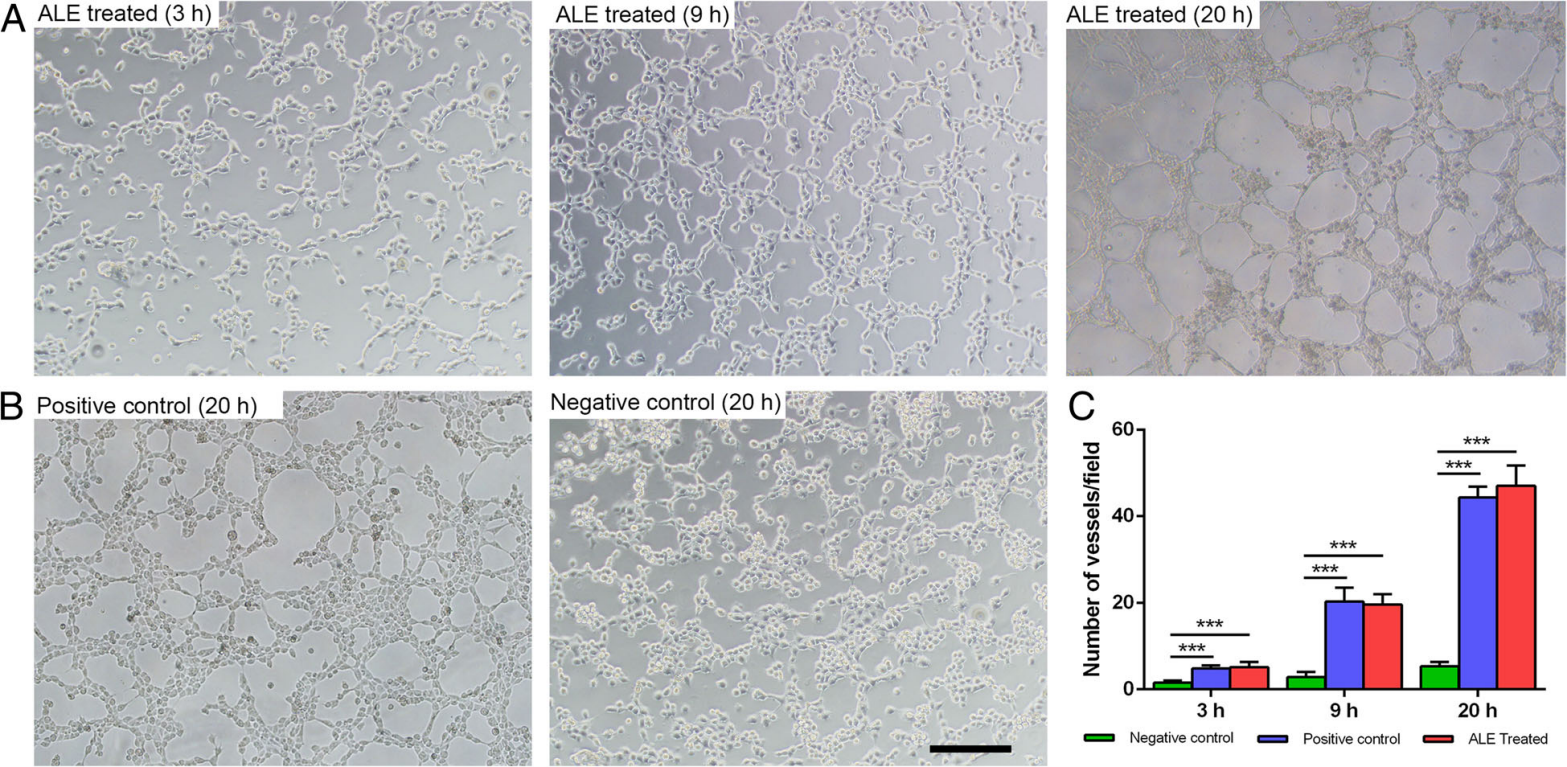
Tubule formation of HUVECs in the presence of ALE. **a** Tubule formation of HUVECs with ALE treatment at different time points. **b** Tubule formation of HUVECs in positive control and negative control groups at 20 h. **c** Quantification of tubular structures in all groups at 20 h. ***P < 0.001. Scale bars = 200 μm

The angiogenic induction ability of ALE was tested by culturing HUVECs in ALE-supplemented medium. In the presence of ALE, tubular structures started to develop at 3 h, and ALE stimulated a time-dependent induction of tubule formation in the assay (Fig. 5a). The production of typical tubular structures was very similar to that in the positive control group at 20 h. However, tubular structures were hardly detected in the negative control group throughout the assay (Fig. 5b). Quantification analysis revealed that the number of tubular structures was significantly higher in both the ALE-treated group and the positive group than in the negative group (Fig. 5c).

**Fig. 6:**
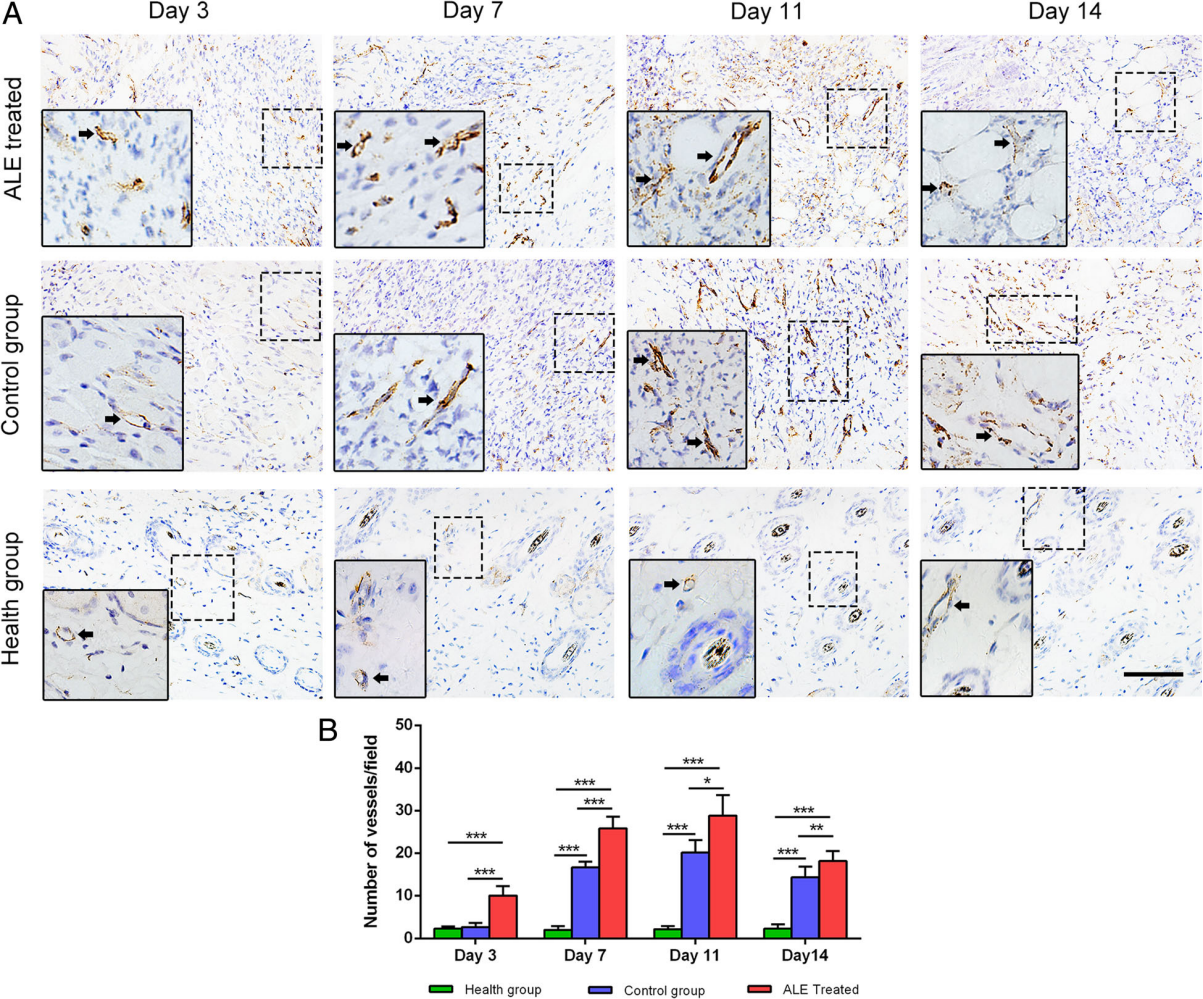
Angiogenesis in vivo. **a** Immunostaining of CD31+ vessels (black arrows) in wounded skins. **b** Quantification of CD31+ vessels. *P < 0.05, **P < 0.01, ***P < 0.001. Scale bars = 400 μm

The angiogenic potential of ALE was further evaluated in a mouse wound healing model. Immunohistochemical staining for angiogenesis marker, CD31, in mouse skin samples revealed that the CD31+ neo-vessels were most frequently observed in both groups from day 7 to day 14 (Fig. 6a), while only a small number of neo-vessels were observed in healthy skins at all time points. Quantification of angiogenesis revealed that the number of CD31+ vessels was significantly higher in the ALE-treated group than in the control group at all time points (Fig. 6b).

### ALE induced adipogenesis in vitro and in vivo

**Fig. 7:**
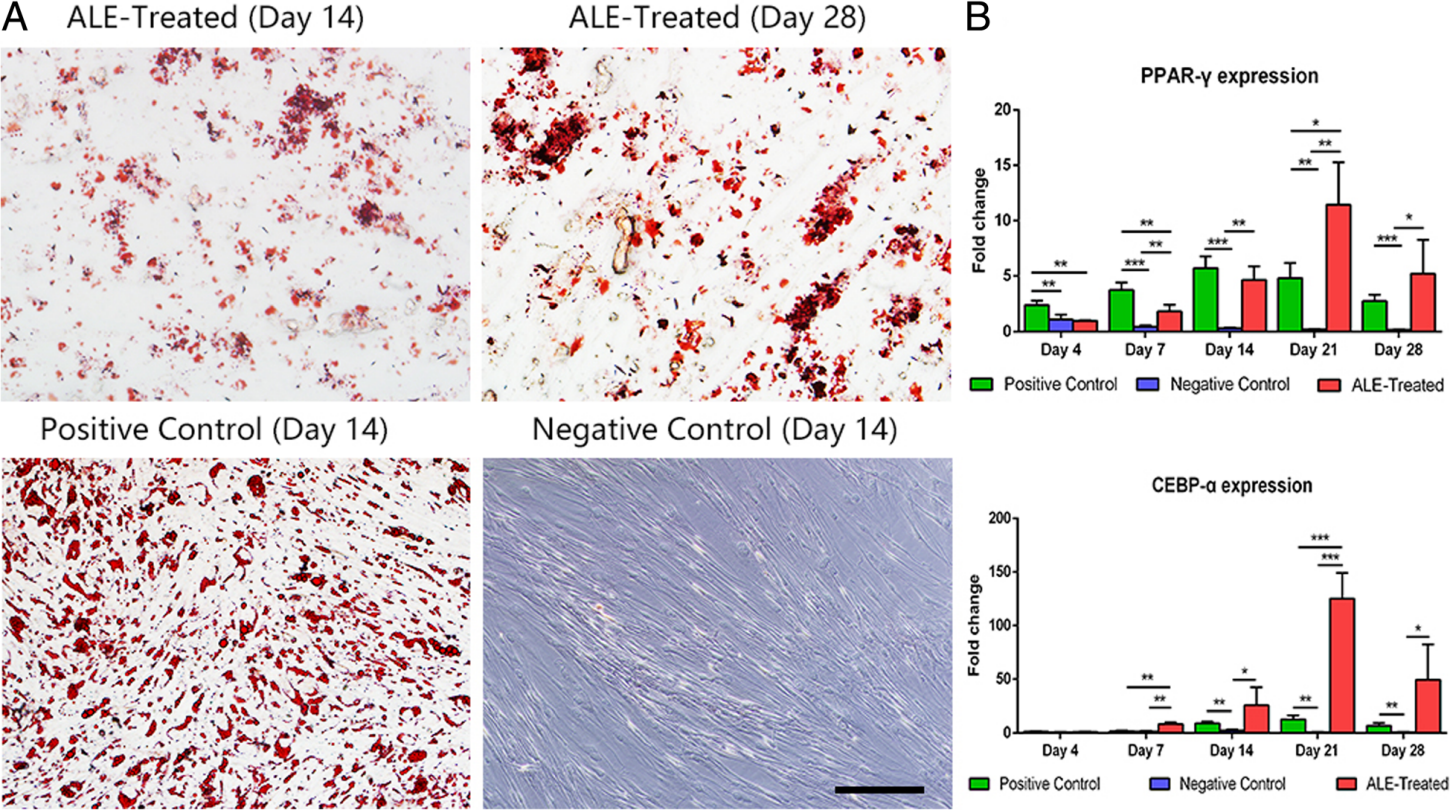
Adipogenic differentiation of ADSCs in the presence of ALE. **a** ORO staining of ADSCs. **b** The relative mRNA expression levels of *PPAR-γ* and *CEBP-α* in ADSCs. *P < 0.05, **P < 0.01, ***P < 0.001. Scale bars = 200 μm

The adipogenic induction potential of ALE was tested by culturing human ADSCs in ALE-supplemented medium up to 28 days. ALE was shown to induce lipid accumulation in ADSCs in the culture, the effect being more extensive when longer culture times were used (Fig. 7a). The relative gene expression of adipogenesis markers, *PPAR-γ* and *CEBP-α*, in ALE-treated ADSCs exhibited a time-dependent increase from days 4 to 21 and a decrease from days 21 to 28. Statistical analysis revealed that the relative expression of the two adipogenesis markers was significantly higher in the ALE-treated ADSCs than in the negative control from days 14 to 28 (Fig. 7b).

**Fig. 8:**
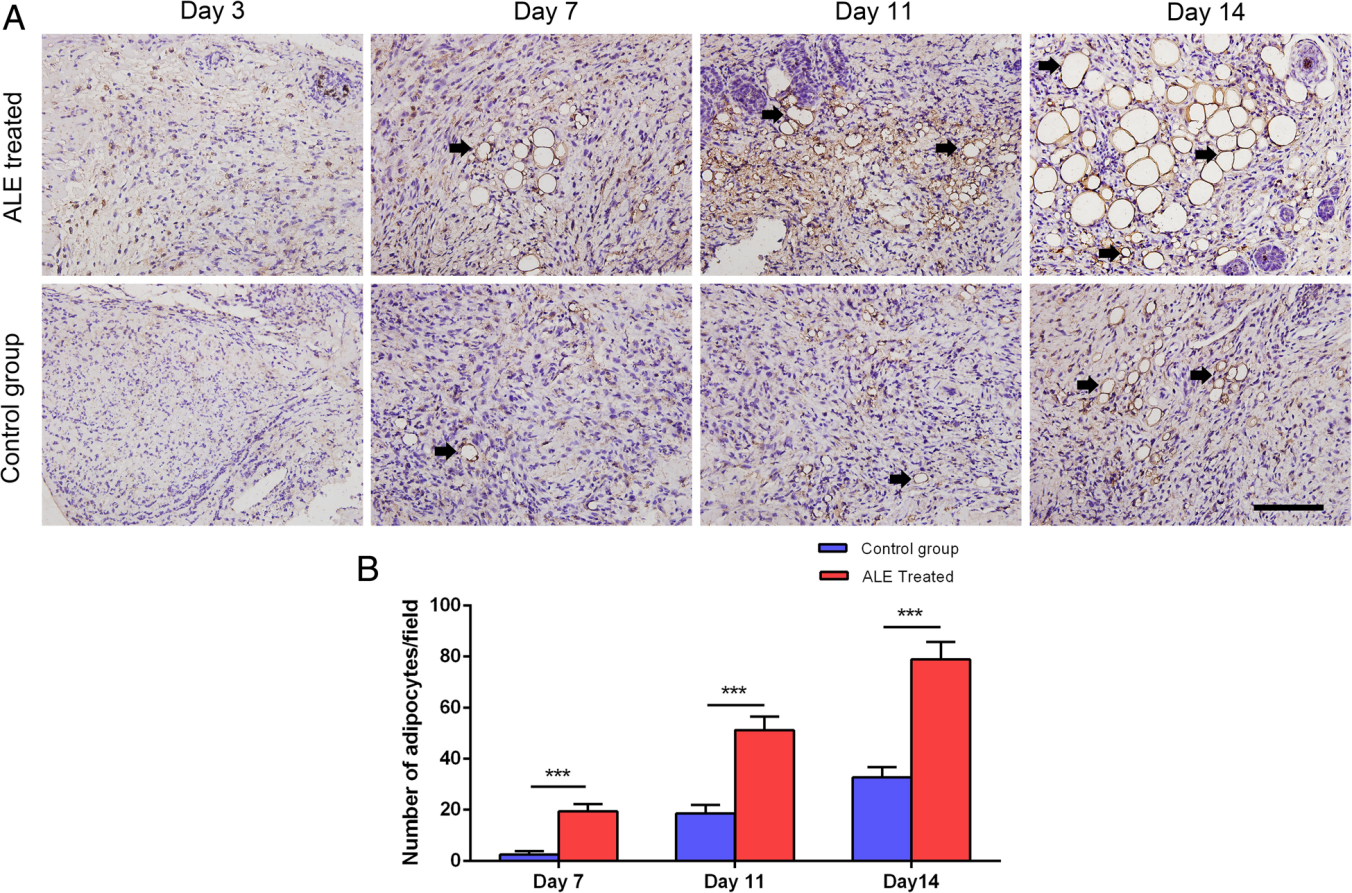
Adipogenesis in vivo. **a** Immunostaining of perilipin+ adipocytes (black arrows) in wounded skins. **b** Quantification of perilipin+ adipocytes. ***P < 0.001. Scale bars = 400 μm

In vivo results revealed that perilipin+ adipocytes could be observed in the skin of ALE-treated wounds at day 7, and frequently observed from day 11 to day 14, while adipocytes appeared scattered in control skins from day 11 to day 14 (Fig. 8a). Moreover, quantification of adipogenesis revealed that the number of perilipin+ adipocytes was significantly higher in the ALE-treated group than in the control group at all time points (Fig. 8b).

## Discussion

Growth factor therapy holds a great promise in regenerative medicine applications. Ideally, therapeutic growth factors should also be cost beneficial, easy to prepare, safe, and reproducible (Table 3). In this study, we prepared a cell-free, growth factor-rich, adipose liquid extract using an easy and rapid method. ALE was shown to induce tube formation of HUVECs and adipogenic differentiation of ADSCs in vitro. More importantly, ALE-treated wounds were more angiogenic, adipogenic, and normalized than the control wounds in mice. Further analysis revealed that ALE, despite the differing processing time periods of the adipose tissue, had similar protein yield, and osmotic pressure values of their liquid extracts were within the normal physiological range. Cell viability assay demonstrated that ALE did not affect cell viability at different processing time points. Thus, 1 min processing of adipose tissue is time-saving and convenient, and therefore, recommended as a standard ALE isolation procedure.

In this study, we demonstrated that the liquid extract from human adipose tissue has a unique capacity of inducing angiogenesis and adipogenesis. In vitro, ALE was shown to efficiently induce tubule formation of HUVECs. Interestingly, ALE treatment exerted a similar proangiogenic ability compared to that of the positive group (VEGF and bFGF treatment) in vitro. The strong proangiogenic ability of ALE could be explained by the presence of multiple angiogenic factors, and a combination of these factors likely acts synergistically to induce angiogenesis [32, 33]. Angiogenesis is a critical process of wound healing [34], and the development of wounds is mainly attributed to the deficiencies of endogenous growth factors [35, 36]. The progression of angiogenesis is controlled by a wide variety of angiogenic factors, which may play important roles in endothelial cell recruitment, proliferation, and differentiation [37]. These processes rely on the cross-talk between various angiogenic growth factors to initiate neo-angiogenesis, which may account for the early angiogenic response of ALE treatment in the skin wounds. Consequently, the enhanced angiogenesis in ALE-treated wounds may have contributed to the healing of such wounds. ALE was shown to induce adipogenic differentiation of ADSCs less than 14 days after treatment. The formation of adipocytes from precursor stem cells involves a complex and highly orchestrated program of gene expression [38]. Important adipogenic genes, *PPARγ* and *CEBPα*, were significantly increased in the ALE-treated ADSCs compared with those in the control. Adipogenesis was further observed in the skin of wounds after ALE treatment, indicating that ALE is efficacious in inducing adipogenesis. Interestingly, ALE induced a higher *PPARγ* and *CEBPα* expression in ADSCs compared to the standard adipogenic medium. Adipogenic medium is consist of five adipogenesis-related contents (glutamine, insulin, 3-isobutyl-1-methylxanthine, rosiglitazone, dexamethasone), while ALE contains 25 adipogenesis-related factors. Among all these factors, adiponectin, sulfotransferase, and aldehyde dehydrogenase have been proven to contribute to adipogenesis [39–41]. Therefore, we speculated that the presence of multiple adipogenesis-related factors and a combination of these factors may likely act synergistically to induce adipogenesis, leading to higher adipogenic marker expression in ALE-treated cells compared to the standard adipogenic medium. Moreover, a previous study by Uriel et al. showed that secretory factors of cultured adipose tissue induced higher *PPARγ* and even fourfold higher triglyceride accumulation of ADSCs compared with adipogenic medium treatment [11]. Although they did not clarify the exact mechanisms underlying this phenomenon in their study, it could be demonstrated that adipose-derived factors is efficient for reproduction and modeling of natural adipogenesis. However, more work should be done to clarify the exact mechanisms why higher adipogenic markers were expressed in ALE-treated cells compared to standard adipogenic medium in our future study. Collectively, the proangiogenic and proadipogenic features of ALE suggest that this agent could be used for overcoming the challenge of vascularization and further adipogenesis in soft tissue engineering. Proteomic analysis was conducted to further evaluate the responsible factors for the angiogenic and adipogenic effect of ALE, and the results revealed that among the 1742 proteins detected, 67 were angiogenesis-related and 25 adipogenesis-related. Thus, we can speculate that the proangiogenic and the proadipogenic effect of ALE is attributed to the presence of a combination of various angiogenesis- and adipogenesis-related factors.

Adipose tissue is a rich and very convenient source of cells for regenerative medicine therapeutic approaches. In the past decade, the therapeutic effects of ADSCs and adipose tissue have been widely studied [9, 42–44]. The underlying mechanisms of these therapies were attributed to the paracrine growth factors released by various cellular components. Recently, adipose derivatives, such as Nanofat and SVF-gel, containing active SVF components, were shown to exert a therapeutic effect on wounds as reported in multiple basic studies and clinical trials [21, 22, 24, 45]. Nevertheless, ALE therapy is different from these cell-based therapies. The mechanical processing of the adipose tissue breaks most of the mature adipocytes as well as many SVF cell components and cell culture process is not included. Therefore, the growth factors in ALE are not cellular secretome components. Recently, adipose tissue was recognized as an endocrine organ and an important source of biologically active substances possessing local and/or systemic action, such as adipokines [46]. Components of the adipose extracellular matrix, such as glycosaminoglycans and proteoglycans possess the ability to bind soluble growth factors and cytokines, which allow the ECM to serve as a reservoir of these biologically active factors [47, 48]. A recent mass spectrometric analysis of adipose ECM revealed that it contains a variety of matricellular proteins and adhesion glycoproteins known to be key modulators of cell functions, as well as a range of growth factors [49]. Therefore, from our study, it is reasonable to speculate that the mechanical emulsification of adipose tissue led to a rapid rise in bioactive factors that were originally stored in cells and ECM of adipose tissue, and these factors were then collected as ALE after centrifugation of the emulsified adipose tissue.

In 2017, our research team reported a novel adipose tissue derivative, SVF-gel [50]. SVF-gel is particularly rich in viable SVF cells and adipose-derived ECM and has been used in the treatment of hypertrophic scars [51], soft tissue defect [52], and chronic wound healing [24] in recent years. Despite the satisfactory therapeutic effect, SVF-gel is essentially a mature adipocyte-free, SVF cell-enriched adipose tissue, and can be only used for autotransplantation. Compared with SVF-gel and other cell-based therapies, the advantages of using ALE can be summarized as follows: (1) ALE does not contain cells and therefore can be assumed to be nonimmunogenic, which may facilitate allogenic or xenogeneic use of ALE in clinics. The therapeutic effect of human ALE on mice wound healing demonstrated in this study may support the xenogeneic use of ALE in the future. (2) The challenges of stem cell-based therapy, such as poor survival of administered cells or potential tumor development, could be overcome by using ALE. (3) Mass production of ALE has become possible, as the popularity of liposuction has increased globally and lipoaspirates can be easily collected from clinics. (4) ALE is prepared from adipose tissue via a purely physical approach without chemical and biological contamination, largely reducing safety and financial burdens. (5) The preparation of ALE is relatively rapid and simple which allows this procedure to be carried out in an operation room during surgery for autologous use. (6) The cell-free and growth factor-rich properties of ALE suggests that ALE could be easily sterilized, stored, transported, and serve as a potential “off-the-shelf” product for clinical use. Despite the advantages mentioned above, there are still several limitations remaining to be addressed before clinical application of ALE. First, the rates of in vitro degradation and inactivation of growth factors in ALE need to be estimated to help optimize in vivo therapeutic strategies concerning treatment doses and frequencies. Second, because ALE was directly injected in the wound bed, the release of growth factors could not be controlled precisely in this study. Therefore, the use of a controlled release system that combines a delivery vehicle and ALE to make the release orderly and continuous would be preferable. Third, in this study, ALE was obtained only from human subcutaneous adipose tissue; further studies on ALE obtained from other types of adipose tissues (e.g., visceral adipose tissue) and adipose tissue from other species might deepen our understanding of these therapeutic agents and would probably extend their applications. Finally, ALE mainly consists of water-soluble components and the study of lipid-soluble components from adipose tissue has been ignored; therefore, studies related to adipose-derived lipid-soluble component would be valuable in the future.

## Conclusion

This study demonstrates a rapid and simple physical method to prepare a cell-free and growth factor-rich liquid extract from human subcutaneous adipose tissue. ALE contains a wide variety of proangiogenic and proadipogenic factors and efficiently induces angiogenesis and adipogenesis both in vitro and in vivo. Thus, ALE could serve as a novel therapeutic agent in regenerative medicine applications.

## Data Availability

The datasets generated during and/or analyzed during the current study are available from the corresponding author on reasonable request.

## Abbreviations

ADSCs: Adipose-derived stem cells)
ALE: Adipose liquid extract
ANOVA: Analysis of variance
ATCC: American Type Culture Collection
bFGF: Basic fibroblast growth factor
CCK-8: Cell Counting Kit-8
CEBP-α: CCAAT/enhancer-binding protein alpha
EGF: Epidermal growth factor
GO: Gene Ontology
HE: Hematoxylin-eosin
HGF: Hepatocyte growth factor
HUVECs: Human umbilical vein endothelial cells
IPA: Ingenuity Pathway Analysis
LC-MS/MS: Liquid chromatography tandem mass spectrometry
LFQ: Label-free quantitation
ORO: Oil Red O
PBS: Phosphate-buffered saline
PDGF: Platelet-derived growth factor
PPAR-γ: Peroxisome proliferator-activated receptor gamma
qRT-PCR: Quantitative reverse-transcription polymerase chain reaction
SVF-gel: Extracellular matrix/stromal vascular fraction gel
TGF-β1: Transforming growth factor-β1
VEGF: Vascular endothelial growth factor
